# Process of diagnosis and treatment of chronic cough in children at primary hospitals

**DOI:** 10.3389/fped.2022.1018924

**Published:** 2022-12-14

**Authors:** Hua-Qin Shen, Yan-Han Zhang, Jing Zhang, Qiu-Ming Sheng

**Affiliations:** ^1^ Department of Pediatric Medicine, Affiliated Zhou Pu Hospital of the Shanghai Health Medical College, Shanghai, China; ^2^ Department of Respiratory Medicine for Children, Shanghai Children's Medical Center Affiliated to Shanghai Jiaotong University School of Medicine, Shanghai, China

**Keywords:** primary hospital, chronic cough, children, diagnosis and treatment process, treatment efficacy rate

## Abstract

**Objective:**

This study aimed to establish a process for the diagnosis and treatment of chronic cough in children suitable at primary hospitals and improve the treatment efficacy rate and improve health economic indicators.

**Methods:**

Children who visited the Department of Pediatrics, Affiliated Zhou Pu Hospital of the Shanghai Health Medical College from January to December 2021 were randomly assigned to the intervention group (*n* = 206), in which the diagnosis and treatment process proposed here was applied, and a control group (*n* = 211) that did not follow the intervention pathway and followed a pathway with the doctors usual practice based on his/her previous experience. Patients were followed up and data were collected at weeks 0 (time of enrollment), 2, 4, 8, and 12 to evaluate the efficacy rate and clinical value.

**Results:**

(1) No significant differences were detected between the two groups in baseline characteristics, including gender, age, duration of cough (weeks), history of allergy in children and parents, and smoking of family members living in the same household (*p* > 0.05); (2) During the follow-up, all cough symptom scores of the intervention group were lower than the control group. Additionally, at week 12, the treatment efficacy rate of the intervention group (91.70%) was significantly higher than the control group (69.20%) (*p* < 0.05); (3) The quality of life of children in both groups at week 12 was improved compared to the first visit. However, the total score of the intervention group was significantly higher than the control group (*p* < 0.05); (4) At week 12, the referral rate was significantly lower in the intervention group (11.17%) than in the control group (21.33%); (5) The intervention group was better than the control group for the mean monthly medication costs, number of days on errors in childhood, and number of days mistakenly worked by family members at week 12 (*p* < 0.05).

**Conclusion:**

The current process of diagnosis and treatment of chronic cough in children at primary hospitals can improve the effective diagnosis and treatment rate, the quality of life, and other parameters, with good effectiveness and feasibility.

## Introduction

Chronic cough in children refers to the predominant or only clinical manifestation of cough for more than 4 weeks and no obvious abnormalities on chest x-ray (1). The prevalence of chronic cough in Chinese children is 7.67% (2). Cough symptoms are among the most common reasons for hospital visits in children (3). Usually, chronic cough in children is not timely treated, which can contribute to disease progression, causing great health and economic burdens. Approximately billions of dollars are globally spent annually for the diagnosis and treatment of chronic cough (4). In Australia, children with chronic wet cough spend an average of up to AU $1339 per capita per month attending the respiratory clinic (5).

The European Respiratory Society recommends using a standardized diagnostic and treatment process to assess and manage chronic cough in children, improve the overall level and efficiency of care for clinicians, make as early and standardized diagnoses and treatment as possible, and reduce healthcare expenditure (6). The results of a meta-analysis suggested that the use of a standardized process for the presentation and management of chronic cough in children can significantly improve clinical outcomes (7). Chang et al. have demonstrated that the effective rate of treatment increased by 24.7% when a standardized process was applied to evaluate and manage children with chronic cough (8). Another Australian study also found an 18% increase in cough relief in patients using a chronic cough management process compared to the control group (9). Therefore, the establishment of a standardized diagnosis and treatment process for chronic cough in children is essential for the clinical practice of pediatricians, and to improve the efficiency and accuracy of diagnosis and treatment. However, the 2013 guideline on diagnosis and treatment of chronic cough in children developed in China is still used to manage these patients (1), which is technically demanding, and most examination items, including children's lung function, bronchoscopy, and 24-h esophageal pH monitoring, have not been widely performed in primary hospitals, thereby significantly reducing its operability in primary pediatric medicine. Additionally, the practical application of this guideline is limited and affects the standardized diagnosis and treatment of chronic cough in children, which might also lead to misdiagnosis and misclassification or blind referral in primary hospitals.

The most common causes of chronic cough in Chinese children include cough variant asthma (CVA), upper airway cough syndrome (UACS), and post-infection cough (PIC). The overall incidence of these three causes is 88.39% of all childhood chronic cough etiologies (10). Herein, we focused on these three common causes of chronic cough in children based on the energy level characteristics of primary hospitals to establish a diagnostic process suitable for primary hospitals. Our results might supplement the limited primary pediatric medical resources, allowing effective diagnoses and treatments of these common etiologies at the primary level, and improving the management of difficult and complex cases. The role of the Tsukuba Basic Hospital as the “first line of defense” to citizens’ health was also demonstrated in the present study.

## Manuscript formatting

### Process design

This study was performed using a modified Delphi method by de Kleuver et al. (11, 12). To review the primary literature, eight fields of chronic cough in children were considered: the composition of chronic cough etiology in Chinese children using multicenter studies (10); recommendations for the diagnosis and treatment of children with rhinosinusitis (13); guidelines for the diagnosis and treatment of chronic cough in Chinese children (1); expert consensus on the diagnosis and treatment of chronic wet cough in Chinese children (14); management pathways or algorithms for use in children with chronic cough (CVD) (15); guidelines for the diagnosis and treatment of chronic cough in adults and children (6); guidelines and expert consensus on chronic cough in children (16); and the use of medical history as a diagnostic tool for chronic cough in children (17). To summarize the above literature, we described the process of diagnosis and treatment of chronic cough in children, combined with the current pediatric situation in primary Chinese hospitals. The study group began by developing a process for the diagnosis and treatment of chronic cough in children at primary hospitals.

After the initial diagnosis and treatment process was developed, we invited 17 physicians with senior job titles and more than 10 years of pediatric experience, including 5 respiratory physicians from children's specialty hospitals, 5 pediatricians from general hospitals, and 7 pediatricians from community health service centers. We performed two rounds of expert anonymous correspondence on the initial formulation of the process. Experts should evaluate the accuracy, feasibility, applicability, efficacy, and safety of the process, and make suggestions for revisions. Then, we collected the revisions and feedback, followed by the second round of correspondence. Next, we revised the process again based on the evaluations and opinions of experts. Finally, all experts unanimously adopted and finalized the proposed process of diagnosis and treatment of chronic cough in children at primary hospitals proposed by our study group.

After the development of the process, a certain number of children with chronic cough were to be recruited, simple randomization was performed using a random number table method, children who applied the “process of diagnosis and treatment of chronic cough in children in primary hospitals” was set as the intervention group, children who used previous experience treatment were set as the control group, and regular follow-up was conducted to evaluate the clinical efficacy and health economic indicators of children and to further verify the effectiveness of the process.

### Study subjects

#### Inclusion and exclusion criteria

Inclusion criteria: ① age 1–14 years; ② patients with a persistent cough for more than 4 weeks from January to December 2021 attending the pediatric outpatient clinic of the Affiliated Zhou Pu Hospital of the Shanghai Health Medical College; ③ parents signed the informed consent. Exclusion criteria: ① common respiratory diseases including classical asthma, bronchopulmonary malformations, chronic lung diseases (i.e., bronchiectasis, cystic fibrosis, bronchopulmonary dysplasia, and bronchiolitis obliterans), hematological diseases, immune deficiencies, congenital heart diseases, gene chromatin abnormalities, and neurological diseases; ② lost during follow-up for at least one reason. This study was approved by the ethics committee of the Affiliated Zhou Pu Hospital of the Shanghai Health Medical College (Ethics number: 2020-c-074).

#### Sample size computer randomization

Sample size calculation was performed using Pass 15 software. This study was divided into an intervention group that used the diagnosis and treatment process of chronic cough in children and a control group that used previous clinical experience. Regarding the conclusions of relevant foreign studies (8, 18, 19), we assumed that the treatment efficacy rate of the intervention group was 90%, the control group was 70; that the variance between the two groups was similar; two-sided test; *α* = 0.05; sample size ratio of 1:1 between the two groups; and a test power of 1 − *β* = 90%. A total of 94 patients were required to be included in the intervention and control groups. After considering a loss to follow-up rate of 20%, at least 118 patients were required in the intervention and control groups. The enrolled cases were simply randomized using the random number table method. The intervention and control groups were divided into 2 groups of physicians with 3 physicians in each group and were independent of each other, in which the physicians in the control group delayed the diagnosis and follow-up with previous experience, and the physicians in the intervention group used the process established in this study to make the diagnosis and follow-up.

### Baseline data acquisition

To complete the baseline data collection at the time of enrollment, the included cases should present the following data: (1) basic information including gender, age, duration of cough before enrollment, history of children and family allergies, and smoking history of same residents; (2) the cough symptom score (20) was used to assess illness severity and the effects of cough on sleep and daily activities, including daytime and nocturnal cough symptom scores (Table 1). The cough symptom score was the result of the daytime cough symptom score plus the nocturnal cough symptom score. Higher scores indicate a more severe cough. (3) The parental cough-specific quality of life questionnaire (PC-QOL) (21–24) addresses physical, psychological, and social dimensions using 27 questions. The 7-point Likert type scale was used for each dimension, and the total score of the questionnaire was the result of the sum of the scores of the 3 dimensions (score range: 3–21 points). In this case, higher scores reflect less frequent coughing, fewer concerns, and higher quality of life.

**Table 1:** Cough symptom score sheets.

Daytime	Night-time
0 = No cough during the day	0 = No cough during the night
1 = Cough for one short period	1 = Cough on waking only
2 = Cough for more than two short periods	2 = Wake once or early due to cough
3 = Frequent coughing, which did not interfere with usual daytime activities	3 = Frequent waking due to coughs
4 = Frequent coughing, which did interfere with usual daytime activities	4 = Frequent coughs most of the night
5 = Distressing coughs most of the day	5 = Distressing coughs preventing any sleep

### Clinical follow-up

**Figure 1:**
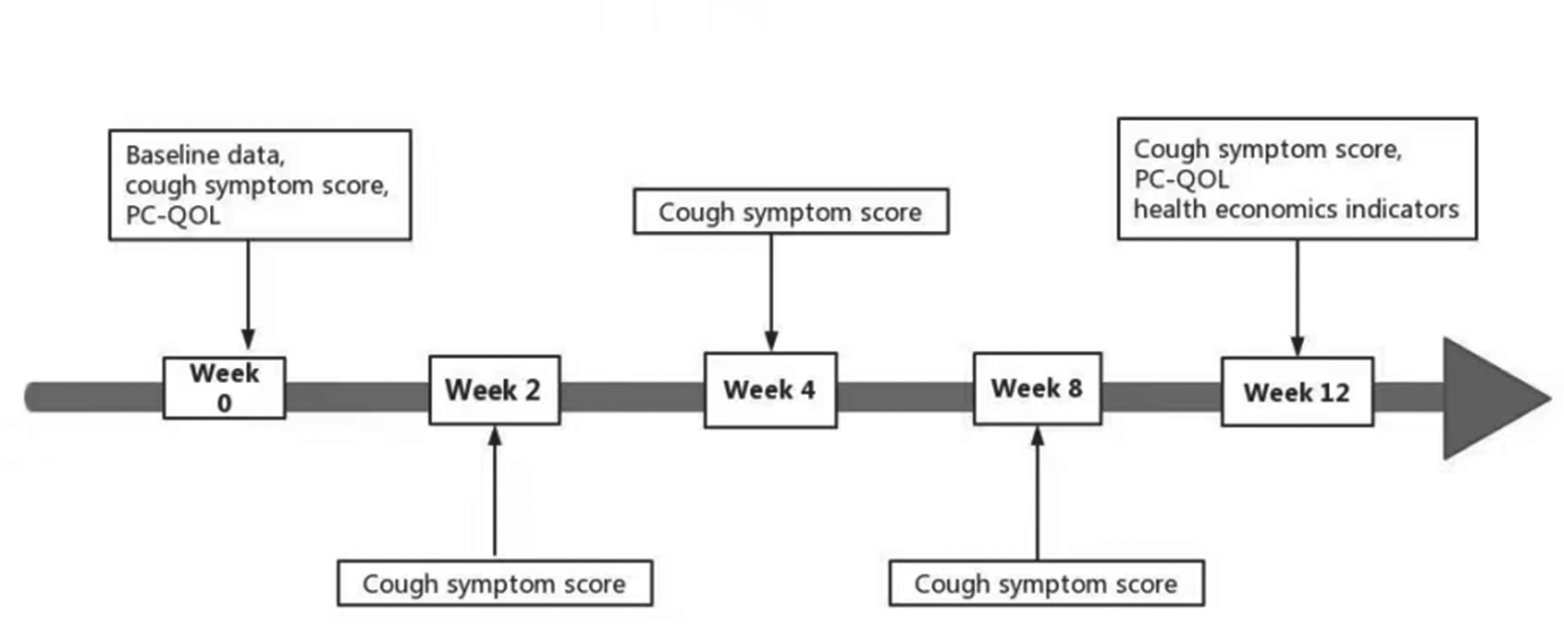
Outcome indicators during follow-up.

Children were first included in the present study if they completed baseline data acquisition at enrollment (week 0). At weeks 2, 4, and 8, outpatient follow-up visits were conducted and the cough symptom score assessments were performed. At week 12, telephone follow-up was conducted and data collection for cough symptom scores, PC-QOL score, and health economic indicators was completed (Figure 1). The health economic indicators included “the average cost of required medications per month due to chronic cough”, “days children delayed school” and “days parents delayed to return to work (because their child is ill)”.

### Outcome indicators

#### Main indicators

The primary outcome measured was the treatment efficacy rate of chronic cough in children: treatment response rate = number of effective patients treated/total number of patients x 100%. Treatment efficacy was defined as a ≥75% reduction in cough symptom score from the first diagnosis at week 12 of enrollment and sustained for more than 3 days.

#### Secondary indicators

(1) PC-QOL;
(2) Referral rate: from the 4th week of enrollment, those whose cough symptom score decreased by <2 points compared with their first referral score and considering poor condition control and referred to a children's specialized hospital: referral rate = number of referrals/total number of patients included in the study and that completed the follow-up x 100%;
(3) Health economic indicators: cost of required medications per month, number of misclassification days in children, and number of days of incorrect work by the family due to the chronic cough.

### Statistical analyses

Statistical analyses were performed using SPSS 21.0. Measurement data are expressed as means ± standard deviations (SDs) and comparisons were performed using two independent samples t-test. Counting data are expressed as cases (%), and the comparison between groups was performed using the *χ*^2^-test or nonparametric test. A *p* < 0.05 was considered statistically significant.

## Results

### Establishment of a process of diagnosis and treatment of chronic cough in children at primary hospitals

**Figure 2:**
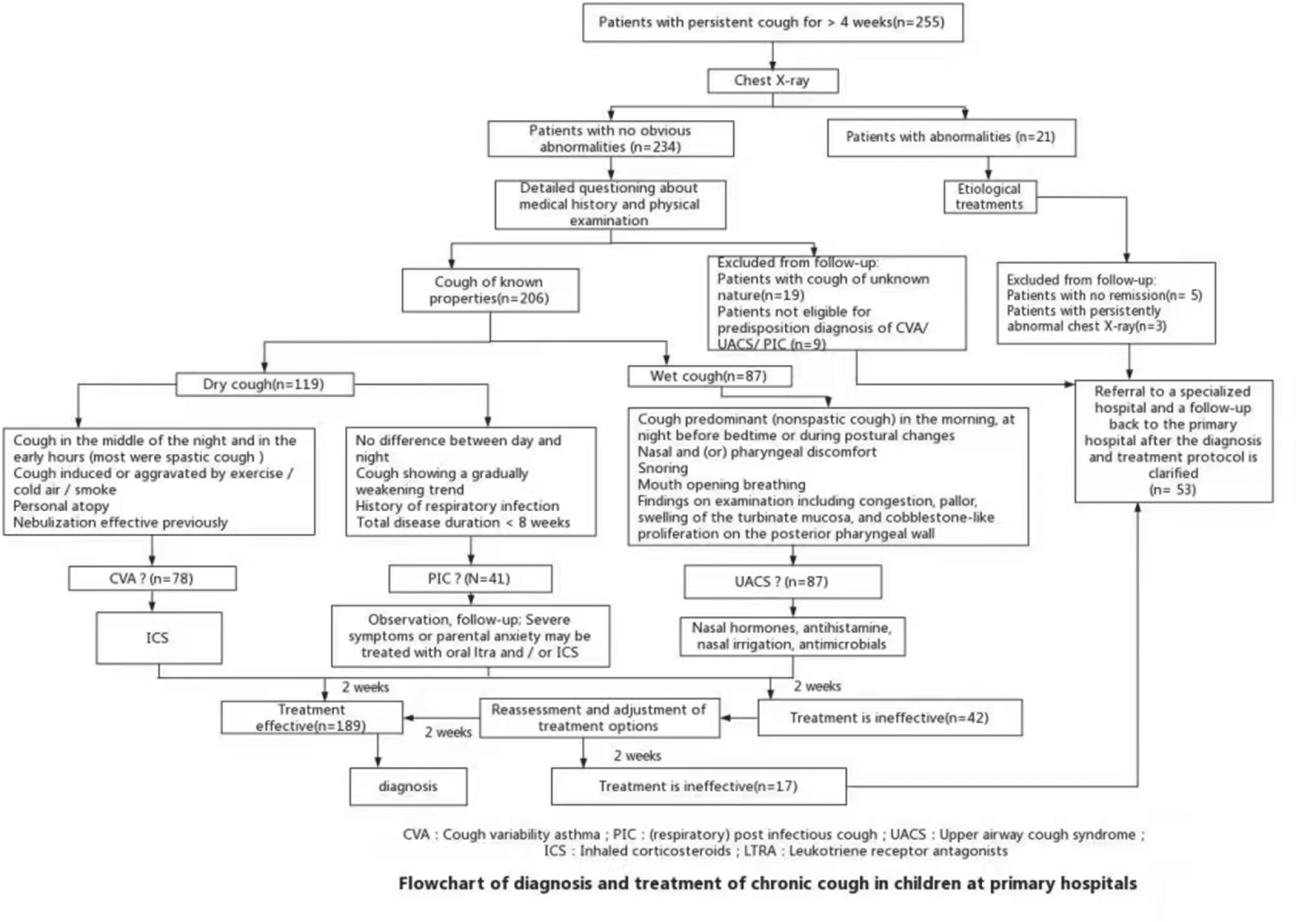
Flowchart of the diagnosis and treatment of chronic cough in children at primary hospitals.

First, children (1–14 years) with cough lasting for more than 4 weeks were approved on chest x-ray according to the diagnosis and treatment process. Patients with abnormal findings were etiologically treated. The patients who did not show obvious abnormalities on the chest x-ray received a detailed history inquiry and physical examination. The medical history included time of onset, nature of cough, phase of predilection, precipitating factors, disease progression, accompanying symptoms, personal history, and previous response to treatment with aerosolized inhalation. The physical examination was mainly focused on the nasal mucosa and pharynx. Next, physicians divided patients into dry and wet coughs based on the nature of the cough, combined with the medical history and physical examination to judge whether the child was prone to CVA, UACS, or PIC. The patients that the nature of the cough could not be established or the predisposition diagnosis was not obtained were indicated to a children's specialized hospital. Third, patients were diagnostically treated based on the predisposition diagnosis. After 2 weeks of treatment during the outpatient follow-up, the diagnosis was confirmed and the original treatment was maintained for those who were treatment responders (i.e., those with a ≥2-point decrease in the cough symptom score from their first diagnosis). Otherwise, re-entry into a second step was required for clinical evaluation and adjustment of the treatment regimen. Then, all enrolled cases were followed up again by an outpatient clinic after 2 weeks, and the treatment responders established the diagnosis and the nonresponders were referred to a children's specialized hospital (Figure 2).

### General information

**Figure 3:**
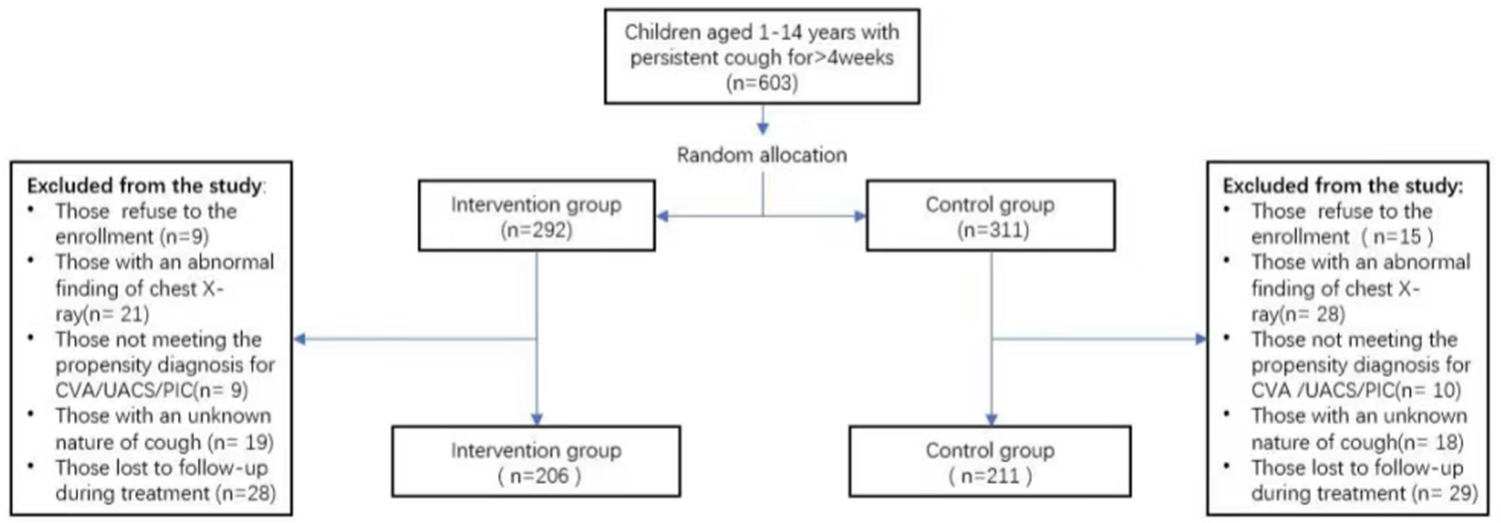
Flowchart of case screening and grouping.

A total of 517 patients were included in this study. Among them, 24 refused enrollment, 19 did not meet the diagnosis of CVA, UACS, or PIC predisposition, and 57 were lost to follow-up at least once. Finally, 206 patients were included in the intervention group and 211 in the control group for analysis. The process of case screening and grouping is shown in Figure 3.

There were no statistically significant differences between the intervention and control groups for gender, age, duration of cough before enrollment, history of childhood and family allergies (i.e., allergic rhinitis, allergic dermatitis, and asthma), and smoking history in same living members (*p* > 0.05; Table 2).

**Table 2:** Basic information and clinical characteristics of patients.

	Intervention group (*n* = 206)	Control group (*n* = 211)	*χ*^2^/*t* value	*p* value
Sex (male)	114 (55.34%)	108 (51.18%)	0.723	0.395
Age				
1–3 years	42 (20.39%)	37 (17.54%)	0.556	0.757
3–6 years	89 (43.20%)	95 (45.02%)		
6–14 years	75 (36.41%)	79 (37.44%)		
Cough duration (weeks)	6.65 ± 1.12	6.32 ± 1.58	1.647	0.100
Allergic diseases in children	161 (78.16%)	153 (72.51%)	1.785	0.182
Parental allergic disease	134 (65.05%)	130 (61.61%)	0.53	0.467
Smoking in same living members	51 (24.76%)	55 (26.07%)	0.094	0.759

### Cough symptom score and treatment efficacy rate

**Figure 4:**
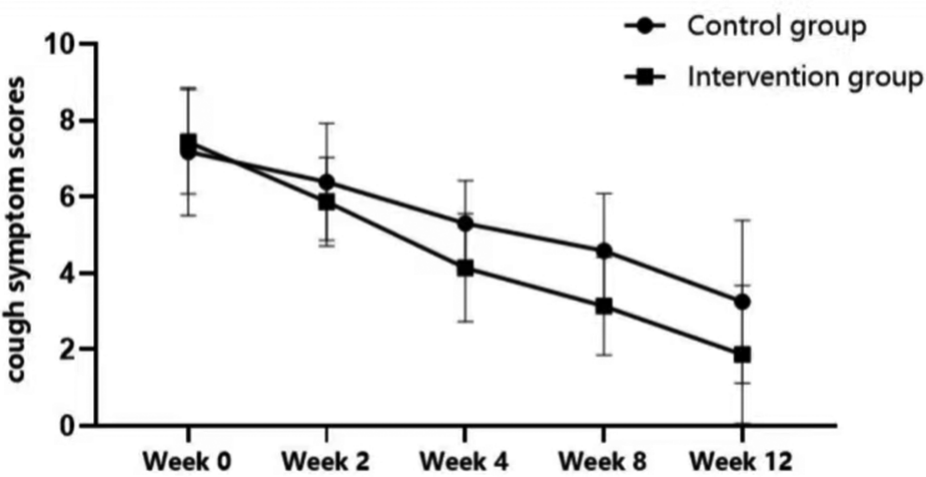
Comparison of cough symptom scores between control and intervention groups.

The cough symptom scores between the intervention and control groups did not statistically differ at the time of enrollment (7.44 ± 1.37 vs. 7.18 ± 1.67; *p* > 0.05). However, the cough symptom scores in the intervention group were significantly lower than the control group at weeks 2, 4, 8, and 12 (*p* < 0.05; Figure 4). The treatment efficacy rate in the intervention group was 91.75% (189/206), significantly higher than the control group [69.19% (146/211)] (*χ*^2^ = 33.562, *p* < 0.05).

### Quality of life scores

The total PC-QOL scores of the intervention and control groups were not statistically different at enrollment (*t* = 0.671, *p* > 0.05). At week 12, the PC-QOL scores significantly improved compared to enrollment (week 0) (*p* < 0.05). Additionally, the PC-QOL scores were significantly higher in the intervention group at week 12 (18. 92 ± 4.02 vs. 15.35 ± 4.42; *t* = 8.619, *p* < 0.05) (Table 3).

**Table 3:** Comparison of PC-QOL scores.

	Total score		*t* value	*p* value
	Week 0	Week 12		
Intervention group	11.28 ± 5.05	18.92 ± 4.02	−16.992	0.000
Control group	10.97 ± 4.37	15.35 ± 4.42	−10.234	0.000
*t* value	0.671	8.619	-	-
*p* value	0.503	0.000	-	-

### Referral rates and health economic indicators

From week 4–12, a total of 23 patients in the intervention group represented a referral rate of 11.17% (23/206), which was significantly lower than the control group [21.33% (45/211)] (*χ*^2^ = 7.887, *p* < 0.05). The intervention group was better than the control group in terms of mean monthly medication costs, days children delayed school and days parents delayed to return to work (because their child is ill) (*p* < 0.05).

## Discussion

Chronic cough is a common respiratory system disease in children (25, 26). It has a complex and diverse etiology and lacks characteristic clinical manifestations (27, 28), which can easily lead to underdiagnosis and misdiagnosis (29, 30). Chronic cough not only affects the quality of life of children and parents and increases the socioeconomic burden (31–33), but might also be accompanied by an underlying chronic respiratory disease (34, 35). The current process of diagnosis and treatment of chronic cough in children is complicated and highly demanding with specialized technology and equipment (1, 16, 36–38), impairing its use for clinical practice in primary hospitals. Thus, the actual use of this process is not high, and the guidance role of primary pediatricians is limited. Additionally, there are clear differences between the etiological distribution of chronic cough between China and other countries. Thus, the process of diagnosis and treatment established abroad might not be fully applicable to the clinical reality of China (9, 29, 39, 40). Based on the above status, at present, most primary pediatricians use personal experience-based treatment and follow-up, which is more casual and has a large variation between different hospitals and doctors, comprehending one of the important reasons why children's chronic cough cannot be effectively diagnosed. Due to a large number of clinical cases, primary pediatricians need a process of diagnosis and treatment of chronic cough in children more suitable to their reality, which might improve diagnosis and treatment efficacy rates, while avoiding blind referral and misdiagnosis.

Herein, we adopted a modified Delphi method to construct a diagnosis and treatment process for chronic cough in children that might be suitable for primary hospitals. We used two rounds of expert correspondence to develop a diagnosis and treatment process for chronic cough in Chinese children based on its three main causes (CVA, PIC, and UACS) to cover the most common causes in the community. Our process was designed to be a diagnosis and treatment approach that matches the competence of primary care providers and physicians, emphasize regular follow-up and evaluation, and is applicable in primary hospitals. Using this diagnosis and treatment process, all symptoms of cough in children significantly improved during follow-up compared to the control group, with a treatment efficacy rate of 91.75%, a decrease in referral to a children's specialized hospital, and significant improvements in quality of life and health economic indicators.

Foreign studies have found that the treatment efficacy rate of using a standardized process for the diagnosis and treatment of chronic cough in children was improved by 24.7% (8) and 18% (9), respectively, compared to the control group. The PC-QOL scores were also significantly improved. Herein, we obtained similar results using our proposed diagnosis and treatment process. The intervention group achieved a treatment efficacy rate of 91.70% at week 12, which was 22.5% higher than the control group. The PC-QOL score was also significantly higher than the control group, and the referral rate decreased from 21.33% to 7.28% of the empirical treatment. These results indicated that the use of the current process improved the awareness of primary pediatricians of the three most common causes (CVA, UACS, and PIC). Additionally, this standardized diagnosis and treatment regimen led to an increased treatment response rate and decreased referral rates. Tangible implementation of the role of primary hospitals in the diagnosis and follow-up of common pediatric diseases has become one of the essential tools to achieve a model of quality care delivery at the doorstep of the treatment of minor illnesses in the community. Meanwhile, the health economic indicators were significantly improved in the intervention group using our current process, suggesting that its use can lead to a more reasonable and efficient diagnosis and treatment of chronic cough in children and reduce the family's economic and life burden.

The current diagnostic process was focused on a detailed medical history inquiry, accompanied by physical examination at key sites, ancillary tests whenever feasible in the community, and diagnostic treatment to make etiologic judgments. Compared to previous processes of diagnosis and treatment of chronic cough in children, the current process has unique advantages. First, it focuses on highlighting the diagnosis of common causes of chronic cough in children. A previous process has discarded the causes of diseases with low incidence (1), since they need special medical examination equipment and are not suitable for diagnosis and treatment in primary hospitals. Combining the results of epidemiological investigation into the three most common causes of CVA, UACS, and PIC (10, 14) for primary pediatric diagnosis and treatment is more suitable for promotion and application in the basic layer. The treatment of children with chronic cough whose condition was not in remission for 4 weeks was not performed and they were not suitable for continued diagnosis and treatment in primary hospitals. Thus, timely referral to children's specialized hospitals was recommended to avoid delays. Second, the current process uses streamlined language to detect the key clinical features and diagnostic treatment options of CVA, UACS, and PIC, facilitating a more rapid memory and mastery by primary pediatricians. Previous processes designed in other countries and outside the text description of clinical characteristics and treatment options are too complicated and are not integrated into the diagnosis and treatment flow diagram of basic pediatricians due to their specific application difficulties. Third, the current process is suitable for primary-level development based on diagnostic treatment with adequate clinical evidence without excessive laboratory tests and medical equipment. This not only compensates for the limited equipment of examination in primary hospitals but also employs safe drugs to treat chronic cough in children to verify or modify the diagnosis with curative effect and alleviate parental concerns, while also improving treatment adherence. Finally, the assessment and treatment form a closed loop to improve efficiency and medical safety. For the treatment of children with no remission of the disease at 4 weeks, using the advantages of the pediatric medical consortium, referral to a site-directed children's specialized hospital, and closed-loop management of the whole diagnosis and treatment process not only ensures medical safety but also improves parental satisfaction with medical services in primary hospitals.

In summary, the process proposed here has some advantages. It fully combines the characteristics of the visited population and the diagnosis and treatment levels in primary hospitals. Additionally, this process captures the common causes of multiple chronic coughs, is concise and refined and convenient for practical application, and is suitable for use in primary hospitals.

However, since this process was only used in the Affiliated Zhou Pu Hospital of the Shanghai Health Medical College, it has geographical limitations, and the study sample size was relatively small. Hence, its effectiveness and feasibility need to be further validated for use in other regions and primary general hospitals.

## Data Availability

The original contributions presented in the study are included in the article/Supplementary Material, further inquiries can be directed to the corresponding author.
